# *“I have never felt so alone and vulnerable” –* A qualitative study of bereaved people’s experiences of end-of-life cancer care during the Covid-19 pandemic

**DOI:** 10.1186/s12904-024-01619-9

**Published:** 2024-12-26

**Authors:** Lara Burton, Silvia Goss, Stephanie Sivell, Lucy E. Selman, Emily Harrop

**Affiliations:** 1 School of Medicine, Cardiff University, Cardiff, UK; 2 Marie Curie Research Centre, Division of Population Medicine, School of Medicine, Cardiff University, Cardiff, UK; 3 Palliative and End of Life Care Research Group, Population Health Sciences, Bristol Medical School, University of Bristol, Bristol, UK; 4 Marie Curie Research Centre, Division of Population Medicine, School of Medicine, Cardiff University, 8th Floor Neuadd Meirionnydd, Heath Park Way, Cardiff, CF14 4YS UK

**Keywords:** COVID-19 pandemic, Bereavement, Cancer, End of life care

## Abstract

**Background:**

COVID-19 drastically affected healthcare services world-wide. In the UK, many cancer services were overwhelmed as oncology staff were reassigned, and cancer diagnoses and treatments were delayed. The impact of these pressures on end-of-life care for patients with advanced cancer and their relatives is not well understood.

**Methods:**

Secondary thematic analysis of qualitative survey and interview data, collected from family members and close friends bereaved by cancer, as part of a national COVID-19 bereavement study (Survey *N* = 156; Interview *N* = 10).

**Results:**

Four key themes were identified: The impact of COVID-19 on contact with patients towards the end of life; Mixed experiences of support for family members; Variable communication quality from health and social care professionals; Prioritisation of COVID-19 and its impact on patient care. Hospital care was perceived more negatively than community care in almost all areas, while support from cancer charities and district nurses was appreciated the most. Almost all participants felt that COVID-19 was detrimentally prioritised over care for their relative/friend.

**Conclusions:**

People bereaved by cancer were uniquely affected by pandemic-restrictions and disruptions to services. As services re-build post-pandemic, improvements in palliative care in hospitals, investment into community care, and ensuring compassionate communication with patients and families must be prioritised, alongside preparedness for future pandemics or similar events.

**Supplementary Information:**

The online version contains supplementary material available at 10.1186/s12904-024-01619-9.

## Background

In many countries, COVID-19 created major disruption to end-of-life (EoL) experiences for those bereaved during the pandemic [1–3], with cancer and palliative care services significantly impacted [4–10]. Pandemic-associated restrictions led to alterations in service provision, as well as high demand and reduced staffing throughout the pandemic [2, 9]. In the UK, during the first three months of the first ‘lockdown’ (March-June 2020), up to 70% of cancer patients received a delayed diagnosis, with potentially treatable cancers discovered at much later stages than usual [4, 5]. Oncology services saw a 40% reduction in chemotherapy attendance and urgent referrals for suspected cancer reduced by 20% compared to previous years [6–8]. These COVID-19 related disruptions contributed to an estimated 1668 excess cancer deaths between weeks 11 to 40 in 2020 (7 Mar 2020 to 2 Oct 2020) as compared to the pre-pandemic period [11]. This occurred alongside restrictions to the provision of EoL care in hospices and care homes and by third sector organisations, with detrimental consequences for patient care [9].

As a consequence of pressures on health and social care and wide-ranging infection control restrictions such as social distancing and restricted visiting, the grief and bereavement experiences of those bereaved during the height of the pandemic were extraordinarily difficult [1, 3, 12–16], with evidence of higher than usual rates of prolonged grief disorder [12, 15, 16]. Although pandemic restrictions affected all deaths, not just those due to COVID-19 infection, quantitative evidence suggests different care experiences and grief outcomes relating to cause and place of death. Some studies report higher levels of grief and other psychological conditions amongst those bereaved by COVID-19 compared with ‘natural’ (e.g. illness) but not ‘unnatural’ causes of deaths (e.g. suicides, homicides or accidents) [16–18], with the unexpected nature of these deaths identified as an explanatory factor [16, 18]. In the Bereavement during Covid-19 (BeCovid) study, which investigated experiences of pandemic bereavement in the UK, there were no statistically significant differences between COVID-19 and non-COVID-19 deaths; however, worse grief outcomes were associated with related factors such as unexpected deaths and deaths in hospice or hospital [14, 15]. Unexpected deaths, deaths in hospital or care home and COVID-19 deaths also increased the likelihood of poorer experiences at the EoL, including perceived lack of support from healthcare professionals and social networks [13].

Palliative and EoL care is a vital element of cancer management for patients and relatives, supporting a positive EoL experience, whilst also preparing friends and family for the bereavement process that is to come [19]. Disruptions to cancer and palliative care services and their clinical implications during the pandemic have been well described, including the experiences and views of healthcare staff and of patients [2, 5, 20, 21]. However, less is known about the specific experiences of cancer-bereaved relatives/friends during the pandemic and the associated emotional and psychological impacts [21]. While some positive caregiver experiences have been observed relating to enhanced family-time and coping [22–25], the small number of studies available have more commonly reported negative experiences amongst current [24–26] and bereaved [22, 23] caregivers. These include reduced social support for family caregivers [22, 24], detrimental impacts on caregiver wellbeing, quality of life and perceived efficacy [23–27] and concerns over the quality of care and treatment provided to the patient and the infection risks posed by COVID-19 [23, 24]. The need for families to have a clear understanding of their family member’s condition and declining health, to stay connected with them in the final weeks/days of life and to have the opportunity for final contact before they died has also been described [27].

This secondary analysis of data from the BeCovid study aimed to provide a focused qualitative exploration of the lived experiences of people whose friends/relatives died of cancer during the COVID-19 pandemic, adding to the limited evidence available on this bereaved population, to inform post-pandemic cancer care across settings and preparedness for future pandemics.

## Methods

### Study design

Secondary, qualitative analysis of survey and interview data collected as part of the BeCovid study, a longitudinal, UK-wide mixed methods study exploring the experiences of people who were bereaved during the first two waves of the COVID-19 pandemic [1, 3, 13–15]. Primary analysis of survey data to date has described the challenges of pandemic bereavement, risk factors associated with poor EoL and grief outcomes, and the support needs of the bereaved population [1, 3, 13–15]. The methods and results reported here have followed the COREQ criteria for reporting qualitative research [28], and where relevant the recently published guidance for reporting Reflexive Thematic Analysis [29].

### Participant recruitment and data-collection: primary study

The BeCovid study collected data, via online surveys (quantitative and qualitative data) and qualitative telephone interviews. Survey data were collected at four time points: baseline (28th August 2020 to 5th January 2021) and 7, 13 and 25 months after bereavement [15]. Questionnaires were distributed online through the Jisc software and posted on an easily accessible, study specific website (with paper copies available on request). Surveys consisted of non-compulsory open and closed questions about EoL and bereavement experiences (supplementary file one).

A convenience sample of participants completed the survey, recruited via social media (Facebook, X previously known as Twitter), bereavement support services and organisations representing minority ethnic groups. Semi-structured follow-up interviews were conducted with a sub-sample of survey respondents who consented in the first survey to be contacted about taking part in an interview. Interview participants were purposively sampled to reflect key characteristics (cause of death, relationship to deceased, gender, ethnicity, sexual orientation). Twenty four people took part in a first interview and 15 took part in a follow up interview, although only first interviews were used for this analysis which was focused on describing end-of-life experiences. First interviews were conducted between April and August 2021, and lasted between 38 and 98 min (mean time: 58 min). Interviews took place via telephone or Zoom and were audio recorded and professionally transcribed verbatim (Supplementary file two, topic guide). Interviews were conducted by four female healthcare researchers, two of whom were also palliative care professionals. All interviewers were experienced in conducting interviews and/or supportive conversations on sensitive topics such as dying, grief and bereavement, which helped them to manage the upsetting nature of some of the interviews. This included adopting self-care strategies such as taking breaks after interviews and allowing space between interviews. The team had regular debriefs to discuss the interviews and checked in with interviewers after each interview. The interviewers also sent a follow up e-mail to participants after the interview to thank them, provide information on further support options and to check on their wellbeing.

Ethical approval was granted by Cardiff University School of Medicine Research Ethics Committee (SMREC 20/59). Participants consented to anonymised data being used in further research.

### Secondary analysis: sampling participants and data

This analysis describes responses relating to EoL experiences from participants who identified ‘cancer’ as the cause of death of their relative/friend. Qualitative survey data were extracted from free text responses of questions A5-B8 of the baseline survey. Transcripts of the ten interviews conducted with cancer-bereaved survey participants were screened to identify sections relating to cancer journeys and EoL care experiences. Relevant extracts were thematically analysed, and results integrated with those from surveys.

Eligibility criteria reflect those of the main study with the addition of cancer being the cause of death. The initial BeCovid baseline survey was open to all adults aged 18 and older who had lost a family member or close friend in the UK after social distancing measures in the UK had been implemented (16th March 2020). For the purpose of this secondary data analysis, only those who indicated that their family member/friend’s cause of death had been cancer were included, with no further exclusion criteria.

### Analysis

Survey and interview data were analysed separately using NVivo 12 Plus [30], starting with the free-text survey data. The data was analysed thematically, involving line-by-line coding and generation of descriptive and analytical themes across the survey data-set [31, 32]After initial reading of survey responses, first author LH coded each survey in turn, generating codes which closely described individual participant experiences or perspectives. LH then created broader codes which meaningfully described shared or similar experiences across participants, and organised and connected these under higher-level analytical categories. This was an iterative process in which the researcher moved backwards and forwards between the data and analytical concepts, meeting regularly with project supervisors and co-authors (EH, SG, SS) to discuss emerging themes and preliminary and final versions of the coding framework.

The same approach was repeated for the interview transcripts. LH then combined the descriptive and analytical themes from the survey and interview frameworks using tables in Microsoft Word. These were further discussed and refined by the team to create a final set of themes and subthemes. These were largely derived from the survey analysis, with the more detailed insights developed in the interview data expanding and elaborating on survey themes. Final themes were fully described and inter-connected in the narrative of results which was drafted by LH and critically reviewed by all authors. Throughout this process the different knowledge and backgrounds of the team informed our discussions and interpretations of the meaning and significance of the data. Whereas the project supervisors/co-authors were experienced grief and palliative care researchers, with varying degrees of knowledge of the full study data-set and findings, LH was a fourth year medical student with practical healthcare expertise and experience of working with patients and families, but less familiarity with the academic literature or wider study.

## Results

### Participant characteristics

Among the BeCovid baseline participants (*n* = 711), 156 had experienced a cancer bereavement (21.9%; *n* = 156/711) and were included in this secondary analysis. Most of these cancer-bereaved survey respondents were women (*n* = 129, 82.7%) and did not identify as belonging to a minority ethnic group (*n* = 149, 95.5%). The mean age of these survey participants was 50 and most had lost either a parent (*n* = 67, 42.9%) or a partner (*n* = 51, 32.6%). Out of the 10 interview participants (who had also participated in the survey), 8 were female, 2 belonged to a minority ethnic group, 5 had experienced the death of a partner. Their mean age was 54. Home was the most frequent place of death reported in the surveys (*n* = 80, 51.3%), followed by hospital (*n* = 37, 23.7%). In the interview cohort, there was a higher proportion of deaths in hospices (60%; *n* = 6/10) as compared with the survey cohort (19.9%; *n* = 31/156). About half of participants considered that their relative was ‘expected’ to die around the time that they died (survey participants: 47%; *n* = 74/156; interview participants: 50%; *n* = 5/10) (Tables 1 and 2).

**Table 1:** Characteristics of the bereaved study participants (separately for survey and interview participants)

		survey participants (*n* = 156)		interview participants (*n* = 10)	
**Age (years)**					
	*Mean [Median]*	*50.2 [51]*		*54.4 [56.5]*	
	*SD*	*12.2*		*8.7*	
	*Min-Max*	*22–90*		*39–68*	
		**N**	**%**	**n**	**%**
**Gender**					
Female		129	82.7%	8	80%
Male		25	16.0%	2	20%
Non-binary		1	0.6%	-	-
Prefer not to say		1	0.6%	-	-
**Ethnicity**					
From a minority ethnic background		7	4.5%	2	20%
Not from a minority ethnic background		149	95.5%	8	80%
**Highest qualification**					
None or GCSEs*		14	9.0%	1	10%
A-level or Apprenticeship or ONC**		27	17.3%	-	-
HND or University Degree***		115	73.7%	9	90%
**Sexuality**					
Heterosexual/straight		125	80.1%	6	60%
LGBTQ+		13	8.3%	2	20%
Unanswered		18	11.5%	2	20%

*GCSE = General Certificate of Secondary Education for 15 and 16 year olds in the UK

** A Levels = Advanced Level subject-based qualification for students in the UK aged 16 and above; ONC = Ordinary National Certificate (equivalent to A Levels)

*** HND = Higher National Diploma (vocational qualification provided by higher or further education colleges in the UK)

**Table 2:** Characteristics of the person who died for both survey and interview participants

		survey participants (*n* = 156)		interview participants (*n* = 10)	
**Age (years)**					
	*Mean [Median]*	68.7 [70.0]		61.4 [58.0]	
	*SD*	14.1		17.7	
	*Min-Max*	2–97		38–96	
		**n**	**%**	**n**	**%**
**Age (grouped)**					
< 20		1	0.6%	N/A	N/A
20–39		4	2.6%	1	10.0%
40–59		32	20.5%	5	50.0%
60–79		87	55.8%	2	20.0%
≥ 80		32	20.5%	2	20.0%
**Relationship to the bereaved person**					
Child		2	1.3%	1	10.0%
Sibling		7	4.5%	2	20.0%
Parent		67	42.9%	2	20.0%
Grandparent		9	5.8%	N/A	N/A
Other family		12	7.7%	N/A	N/A
Partner		51	32.6%	5	50.0%
Friend/colleague		8	5.1%	N/A	N/A
**Place of death**					
Care home		6	3.8%	N/A	N/A
Hospice		31	19.9%	6	60.0%
Hospital		37	23.7%	1	10.0%
Their home		80	51.3%	3	30.0%
Other or no response		2	1.3%	N/A	N/A
**Death expected?**					
Yes		74	47.4%	5	50.0%
No		69	44.2%	5	50.0%
Don’t know		12	7.7%	N/A	N/A
No response		1	0.6%	N/A	N/A

### Key themes

Four main themes and eight sub-themes are described relating to the EoL period. The main themes are: (1) The impact of COVID-19 measures on contact with the person who died; (2) Support for family members during the EoL period; (3) Communication with health and social care professionals; (4) Prioritisation of COVID-19 and the impact on patient care (Table 3).

**Table 3:** Overview of qualitative themes

Main themes	Sub-themes
The impact of COVID-19 measures on contact with the person who died	• Restricted contact and communication challenges • (Non)adherence to guidelines
Support for family members during the end of life period	• Informal support networks, isolation, and loneliness • Variable provision of formal support for caregivers
Communication with health and social care professionals	• Lack of communication and information regarding patients • Variable communication quality
Prioritisation of COVID-19 and the impact on patient care	• Challenges accessing care and de-prioritisation of cancer services • Inconsistent quality of patient care

#### The impact of Covid-19 measures on contact with the person who died

A primary source of distress for almost all participants was the impact of COVID-19 restrictions on spending time with their dying relative during the last days or weeks of life.

##### Restricted contact and communication challenges

During the 2020 ‘lockdowns’, care facilities implemented infection control measures which prevented relatives/friends from spending extended time with patients at the EoL. Participants feared they had let down their dying relatives, and felt guilty for leaving them alone for prolonged periods in unfamiliar, vulnerable environments. Visiting restrictions were particularly distressing when they resulted in someone dying alone, especially in hospitals.

*I’m haunted by the fact mum was on her own and must have been terrified*. *She was the matriarch of our family and did everything for us and feel we let her down*. *I can’t move past this*. Bereaved daughter, whose mother died in hospital, survey PID 364.

Even for those able to visit, the frequency and/or number of visitors was heavily restricted. Choosing which family members could visit was perceived as a great injustice, with some families “drawings straws” (PID567) to reduce potential tension.

*…the door to her room was kept shut and nobody came in*. *I was alone with her when she died as they would only allow one visitor*. *I have never felt so alone and vulnerable*. Bereaved sibling, whose sister died in a hospice, survey PID 633.

COVID-19 measures also prevented visiting people at home. Many participants described the impersonal and inadequate nature of ‘visiting’ through a window or sitting two metres apart in gardens. Lack of physical closeness was highly upsetting, with people feeling unable to comfort their relative during their illness and final days of life.

*Basically*, *the restrictions ruined the last months of his life*. *He needed moral support*, *love and companionship*, *which he was unable to get… I couldn’t say goodbye to or hug my own father*. *I’m finding that very difficult to accept*. Bereaved daughter whose father died in a care home, survey PID 304.

People reported variable communication with their dying relatives, something which was particularly important during hospital or hospice stays where visiting was restricted. Communication challenges included reliance on staff members’ assistance (associated with infrequent, short phone calls) or patients’ difficulties speaking e.g. due to tumour progression or declined health state.

*With him not being able to text or use his phone our contact with him was limited to the portable ward phone but as we couldn’t see if he was asleep or anything we often only spoke to him for a few precious minutes*. Bereaved wife whose husband died at home, survey PID 104.

##### (Non)adherence to guidelines

Enhanced shielding guidelines for ‘vulnerable people’ (e.g. cancer patients) to isolate at home, were recommended before whole population restrictions. However, for most people, the wish to spend time together was considered more important than full adherence to regulations. The inevitability of death and the desire to maximise time spent together mostly overrode fear of infection or a moral imperative to follow rules.

*I wasn’t able to see my dad for 4.5 months*. *As soon as I found out he was terminal I visited him and stayed with him as I wasn’t prepared to not see him and provide care and support*, *regardless of covid*. Bereaved daughter whose father died at home, survey PID 413.

Strict guideline adherence was, however, described by a minority of participants. Those who followed shielding and distancing advice very closely described being unaware that their relative’s cancer was terminal or, if they were acting as their relative’s primary carer, being anxious about what would happen if they themselves became unwell. These participants often expressed regret and frustration about this time spent in isolation from one another, while some also lamented the loss of quality of life for the person who died.

*So we went into coping with all that and managing live in the house on our own and what we decided was*, *I was going to completely shield with her*, *although it would have to be in separate rooms and all that sort of stuff*, *in our own protected bubble*. Bereaved partner whose spouse died at home, interview, PID 484.

One interview participant, a healthcare professional, also described her niggling doubts and regrets over whether she might have noticed that her father was sick much sooner had she not stayed away to protect him.

*Just the weeks leading up to him being poorly that I’ve missed*, *if I’d known at the height of Covid that my dad was going to start to show signs of cancer*, *would I have stayed away or would I have I don’t know*, *put gloves and pinnies on and just gone to see him every day? I think the last weeks now I’d love to have back but I can’t *[*crying*]. Bereaved daughter whose father died at home, interview, PID 510.

Lack of clarity and frequent changes to guidelines lead to confusion and frequent, frustrating miscommunications from healthcare providers. People were grateful when health and social care professionals made exceptions to visiting restrictions, allowing more time with the patient. This was not uncommon, especially in the final days/hours of life.

*In general the hospital staff did everything possible in difficult circumstances*. *They lifted a no visitor policy to allow me to stay for the last four days of her life*. *Our three children were also allowed to visit the day before she died*. Bereaved partner whose spouse died in hospital, survey, PID 486.

#### Support for family members during the end of life period

Participants reported that accessing informal and formal support in the weeks leading up to and immediately following the death was especially challenging.

##### Informal support networks, isolation, and loneliness

COVID-19 measures significantly impacted participants’ ability to receive emotional and practical support from family and friends. This was particularly challenging for those whose family did not live nearby, or who lived alone, thus relying on phone calls and messaging.

*I live on my own*, *work from home and don’t really have a support bubble as everyone is with others*. *It is very lonely and often feel abandoned*, *even though I acknowledge no one is at fault*. *I am dreading Christmas as this was always our time and this year*, *I will have no one*. Bereaved daughter whose father died in hospital, survey, PID 516.

Cessation of regular activities and gatherings contributed to the inability to escape from the situation, described by one participant like “being in a capsule”(PID598). Lack of physical comfort was especially hard to cope with and contributed strongly to the feelings of isolation and loneliness that characterised the EoL period for many.

*Zoom does not replace coming together to mourn*. *I still have not been able to give my Dad a hug and it was his Dad who died*, *due to social distancing*. *Bereavement under social distancing is just awful*, *understandable but awful*. Bereaved granddaughter whose grandfather died at home, survey PID 41.

*Yeah*, *I think in hindsight I think it was almost like erm*, *at that time the world shrunk*, *it was like being in a capsule…Erm*, *so that was sort of more or less erm halted. You couldn’t even go out to the*, *I mean I’ve just been swimming this morning which*, *you know*, *I used to do quite a bit*, *I started going to the leisure centre*, *you couldn’t do that cos they were closed. You couldn’t go to the shops*, *just to give you a distraction*. Bereaved daughter whose mother died in a hospice, interview, PID 598.

Despite the challenging circumstances, many people felt well supported by their social networks, thanks to the internet and other communication technology, and appreciated talking and sharing experiences over video call or messages. One bereaved partner valued the enhanced time that she and her partner had together at the end due to the restrictions on in-person socialisation and activities.

*…and I have a very*, *um a one supportive group of friends. So*, *um*, *I was absolutely never short of emotional support*, *whether that was on the phone or on social media…*Bereaved partner whose wife died in hospital, interview, PID 473.

*At the same time Covid restrictions have helped. [Partner] asked that they ‘Stop all the clocks’ and this to a certain extent happened. There was no pressure to go out and socialise. The fact that everyone had to stop seemed somehow appropriate*…Bereaved woman whose partner died at home, survey, PID 484.

##### Variable provision of formal support for caregivers

Formal support for caregivers, as provided by a care setting, HSCP or other organised support, centred around optimising care provision, managing expectations and preparing for death.

Specialist voluntary and community sector cancer services were often considered the most helpful support providers, in terms of both clinical and emotional support (e.g. cancer charity nurses providing home care). With support groups and in-person counselling suspended, telephone support was appreciated and provided helpful preparation for what was to come. Experiences of accessing formal support varied, and reflected the different needs expressed by participants. Those with close, supportive families sometimes declined formal emotional support, while others described “fight[ing] for support” (PID274) as no information was provided.

*But I could’ve accessed them had I wished to*, *easily. Erm*, *I think I come from a generation where we don’t*,* I’ve done so much talking to counsellors and all that kinda thing I don’t know whether’s a good thing or a bad thing…I had the support of my partner who lives with me*, *she and I talked quite a bit and a lot of friends wrote things which were helpful.* Bereaved mother whose son died at home, interview PID 527.

Home care nursing and palliative care staff were considered supportive when they demonstrated concerted efforts to provide personalised care, and actively responded to the concerns, questions or needs of family members. Conversely, reduced interaction left participants feeling under-supported and underprepared in their caregiving roles, making the EoL period and death more traumatic. A desire for better preparation for supporting dying family members was emphasised in the interviews.

*For the week he was home before he died*, *we felt extremely supported by the daily visits (and more when we needed them) of the district nurses and the support of the palliative care team.* Bereaved daughter whose father died at home, survey PID 618.

*…I couldn’t believe how hard it was to find advice how to deal*, *um*, *with somebody with a terminal illness…. And I googled what do you talk … how do you talk to somebody who’s terminally ill… you know*, *I can’t believe (cancer charity) don’t do more on this*, *there was a phrase I read which said what you can do is go to the person and say do you want to talk about how you’re feeling at the moment.* Bereaved wife whose husband died in a hospice, interview PID 310.

#### Communication with health and social care professionals

Maintaining effective and sensitive communication with family members about the patient’s condition and care is another important aspect of caregiver and family support at the end of life. Difficult communication with healthcare professionals regarding patients was a significant shortcoming of pandemic care, which led to frustration and distress due to uncertainty about the health of their relative. The quality of communications also contributed to how prepared people felt for the death, with significant variation across care-settings.

##### Lack of communication and information regarding patients

Challenges in accessing information were almost universal in hospitals, where many participants described repeatedly contacting wards for information or updates. This was frustrating as participants felt that regular updates about their relative’s care and condition should be a core component of care, especially with visiting restrictions in place.

*I had to chase for updates all the time. No fewer than ten people promised updates and to get back to me but I received not one call-back. Not being able to be there on the ground was appalling and traumatic.* Bereaved husband whose wife died in a hospice, survey, PID 391.

Participants frequently received unclear, insufficient, conflicting, or incorrect information, a problem which was compounded by clinical staff being too busy to be contacted. People also described inefficient communication between healthcare teams, becoming frustrated and upset when left to relay information between professionals themselves.

*… two days after dad was transferred to the home and two days before he died (he was only there for four days)*, *his GP rang me demanding to know why his medication had changed at what was wrong with him. I was quite short with her (surely she could have got this information from the hospital or the home without bothering a family member at such a difficult time).* Bereaved son whose father died in a care home, survey, PID 418.

##### Variable communication quality

Dissatisfaction with communication also related to the quality of interactions. Participants felt dismissed when their concerns were not taken seriously (e.g. not investigating new symptoms) or questions not answered clearly, and sometimes perceived a lack of sensitivity when discussing highly emotional issues. Unmet promises, a perceived lack of transparency and feeling hurried contributed to a perceived lack of compassion. Positive communication experiences included being kept well informed, with staff available to address questions and concerns. Open and approachable staff who kept families well informed, took time to listen, and responded with understanding were greatly appreciated. These experiences and qualities were attributed most often to community-based hospice and nursing staff. Experiences with general practitioners were mixed, reflecting both positive and negative experiences.

*The face to face communications with hospice staff were enormously helpful and comforting. I never felt they were rushing me and they always made sure they answered my questions. The booklet ‘When Someone Dies’ was immensely useful. It provided lots of practical and emotional help*, *including about things I wouldn’t otherwise have thought of.* Bereaved son whose mother died in a hospice, survey, PID 597.

*Our GP came out & arranged a community palliative nurse who was marvellous*, *she organised carers & was an enormous support in his last 12 days. So communication was vastly different - from the hospital/oncologist/ specialist nurse being uncommunicative*, *to the community palliative nurse*, *GP*, *district nurses & carers who were absolutely brilliant. We could ring them or they’d ring us.* Bereaved wife whose husband died at home, survey, PID 104.

Telephone communication was generally adequate for short updates, however face-to-face communication was preferred for appointments or detailed conversations. Email updates and hospital letters were largely considered unhelpful; information leaflets and booklets were more positively received. Taking information home to read helped people understand complex information about treatment, illness and EoL which would have been overwhelming to receive verbally.

*We also were given a little book by the [terminal illness support charity] foundation that gave lots of specific details and answered a lot of questions. I found the book very helpful because the subject was very distressing and the book enabled me to just take in small amounts of information as and when I could cope with it*, *and to re-read things that I needed to clarify. I find that when I’m upset*, *it’s often difficult for me to listen and understand fully so the book meant that I could go at my own pace. It also answered some questions that I felt unable to ask the nurses as it was too upsetting.* Bereaved daughter whose mother died at home, survey PID 369.

#### Prioritisation of COVID-19 and the impact on patient-care

Many participants felt that the death of their relative was an unjust consequence of COVID-19. People expressed concerns that non-COVID patients were neglected, as many services were suspended to reduce COVID-19 transmission. Experiences of care during the pandemic, across all care settings, were compared unfavourably with pre-pandemic hopes or expectations of quality EoL care.

##### Challenges accessing care and de-prioritisation of cancer services

Participants reported that accessing the right care and support services was challenging, with some people feeling pushed away due to perceived COVID-19 risk or healthcare professionals not taking relatives’ concerns seriously. Interviews highlighted the view that reduced face-to-face interaction led to patients silently reaching crisis point as new symptoms and deterioration were missed, especially when attempting to access GP care. Healthcare professionals sometimes incorrectly attributed symptoms to COVID-19 infection, which hindered access to necessary care, caused significant upset and contributed to feelings of helplessness.

*… nobody would look at him*, *no healthcare professional would let him anywhere near them*, *they said*, * you know*, *.… you can’t come to any of the hospitals*, *you’ve got COVID. And it went on for weeks.* Bereaved wife whose husband died in a hospice, Interview PID 310.

Fear of COVID-19 was felt to dominate the NHS, with people feeling that cancer services were detrimentally neglected. Suspension of normal services, redistribution of staff and challenges accessing care all contributed to the most significant and widespread cause of anger and distress among survey participants: feeling that COVID was prioritized over cancer.

*I felt like I was having this battle with medics who … and medical people*, *and I think more people have died because of some false*, *false attempt to protect other patients*, *other patients …*Bereaved wife whose husband died in a hospice, interview PID 310.

*Covid is terrible but all normal health care was suspended for months by both the hospitals and the GPs. This meant my family suffered greatly. This must not be repeated*…Bereaved niece whose aunt died at home, survey PID 118.

Many participants wondered whether their relative would have survived under normal circumstances. In the case of new diagnoses, regrets arose around earlier detection, timely help-seeking, face-to-face appointments, and screening. Among patients with known cancer, delays or cancellation of scans, appointments, and/or treatment were common. For many, these delays and cancellations were perceived to be fatal.

*I will never stop wondering whether she might have survived the cancer which took her had Covid not drained the life out of the NHS for all but desperate Covid patients whose lives also hung in the balance.* Bereaved woman whose friend died in a hospice, survey PID 150.

*We were having to deal with [NAME]’s cancer and the fact that as far as I’m concerned they took her off treatment and she would have lived longer*, *and she would have been more comfortable. The fact that she died in discomfort that was unnecessary in this world just*, *I mean I’m not going to go into what’s happening globally*, *but that was really traumatic*. Bereaved wife whose wife died at home, survey PID 484.

##### Inconsistent quality of patient care

Patient care experiences were variable, with some participants expressing significant disappointment with the quality of care provided to their relatives, while others were grateful and satisfied. District and palliative care nurses were frequently praised for providing high-quality care to patients despite COVID-19 restrictions. Participants were grateful for nurse and/or GP visits, appreciating and often choosing home care over hospital admission. Many who were unable to access GP or home care felt neglected and abandoned by the care system. Hospital care ranged from “outstanding” (PID561; PID662) to “poor” (PID575; PID600); negative patient experiences associated with the latter included unnecessary procedures, medication errors and insensitive communication with the patient which was distressing to both patient and family members. Participants sometimes also related these mistakes or shortcomings to their enforced absence and inability to be involved in important care-conversations and consultations, reflecting on the sense of powerlessness and loss of control that they experienced.

*They took her in in a wheelchair and then they phoned me to say she was ready to pick up and I went to pick her up and they were outside with the wheelchair*, *she was there with a bag of drugs and I knew nothing about what had gone on inside and she couldn’t tell me.* Bereaved wife whose wife died at home, interview, PID 484.

*But the memory of him finding out he had terminal cancer whilst he was alone and in pain and then not being able to visit will stay with us forever*, *as will (what felt like) the lack of compassion demonstrated by his GP when we called to ask for pain relief. The private care providers that adult social care engaged were fantastic throughout*, *as were adult social care*, *and many of the community nurses who visited.* Bereaved granddaughter whose grandfather died at home, survey, PID 274.

Inappropriate pain control was a frequent complaint common to hospitals and home care. A perceived lack of pain relief led to ‘traumatic’ thoughts of final hours spent in distress, while too much was reportedly associated with heavy sedation and inability to communicate.

*In her last few days she was unable to speak*, *as she had so much pain relief*, *I wonder if it was too much as she lost her speech…. The hardest part was not being able to see her and my family in the weeks leading up to this*, *and my last chance of seeing her was on her death bed*, *where she was in a lot of pain*. Bereaved granddaughter whose grandmother died at home, survey, PID 678.

Perceptions of healthcare professionals also varied. Poor care attitudes were most commonly attributed to hospital staff, with participants finding them “too busy” (PID616) to provide individualised care for the patient. However, people were usually understanding, blaming systemic pandemic-associated pressures rather than individuals.

## Discussion

This secondary analysis describes the EoL experiences of people bereaved due to cancer during the height of the COVID-19 pandemic. Key findings relate to family members experiences of restricted inter-personal contact and variable healthcare quality. Many of these experiences are not unique to cancer bereavement; loneliness and isolation, visiting restrictions and communication challenges are described across the literature, regardless of cause of death [2, 3, 13–15, 27]. However, important cancer-specific experiences were identified, including the patient and family impacts of extended shielding and isolation requirements and disruption to usual and expected healthcare services during the final months and weeks of illness. Significant variations in the communication and care received across settings are also described in-depth, with important implications for current/post-pandemic and future pandemic care.

### The impacts of restricted social contact on intra-family experiences and support

Reduced time spent together was perhaps the most dominant theme identified in this dataset. Although this affected most people bereaved during the pandemic [3, 27], social contact restrictions disproportionately impacted relatives of cancer patients for whom shielding was recommended due to their impaired immune response. Shielding advice was implemented before population-wide lockdown measures, creating a prolonged period of isolation for those with known cancer and their family and friends compared with bereavements not due to a life-limiting illness. Although many people reported eventually choosing to disregard regulations, the changing nature of these regulations caused frustration. Feelings of regret were caused by missed opportunities to spend time together, diminished quality of life for the person who died, and concerns that early signs of illness were missed due to staying away. Being present at the time of death was important to many people and was often a key motivating factor for those who chose home care for their relative. As has been shown in other bereavement studies, as well as whole-cohort analysis from the BeCovid study, being there and saying goodbye was important for providing a sense of ‘closure’ and helped people prepare for the grieving process [3, 27].

COVID-19 restrictions not only prevented family members from spending time with their sick relative, but also disrupted their informal and usual support networks. The importance of community and social relationships at the end of life and in bereavement is well recognised [15, 33–35]. COVID-related challenges with accessing informal, social support intensified the personal sacrifice and isolation of caring for sick relatives at home, with some caregivers feeling overwhelmed, isolated and unsupported [22, 24]. Unfortunately diminished social support was also matched with reduced access to professional sources of emotional and practical support, further compounding the feelings of burden, stress and uncertainty that are commonly experienced by family caregivers in non-pandemic times [36–38].

### Impacts on patient-care and healthcare quality

The effects of disruptions to cancer care were widely discussed, with many people feeling that cancer services were de-prioritised in favour of treating COVID patients or protecting staff. It is reported that the care of almost two thirds of cancer patients was negatively affected by COVID-19 in the UK [39] and many participants felt that the deterioration and eventual death of their relative was linked to these disruptions. This was perceived as a great injustice and led to significant frustration, anger and anxiety, with many people questioning the inevitability of their bereavement. Unexpected deaths are recognised as a risk factor for poor bereavement outcomes, including prolonged grief disorder, in this and other studies [14–16, 18, 40]. It was striking that only a half of these cancer deaths were ‘expected’ to have occurred around the time that they did. Delaying and cancelling potentially life-saving cancer treatments raises ethical issues around resource distribution and care prioritisation, which must be carefully re-considered post-pandemic, with the negative long-term consequences for bereaved relatives also recognised.

Adequate communication is a core component of healthcare, especially at the EoL, where anxiety and distress levels are high [41]. Poor communication experiences were frequently described by participants bereaved by cancer, as well as by the whole study cohort [3]. Important non-verbal communication is missed in short telephone consultations, which may contribute to perceptions of poor clinician communication skills. However, the primary complaint about virtual consultations related to subtle yet important symptoms being missed or under-investigated, leading to concerns about the accuracy and timeliness of diagnoses and delayed access to treatment, as reported elsewhere from both GP and public perspectives [39, 42]. Whilst the evidence for virtual consultations is generally favourable [43–45], these experiences should not be disregarded when providing virtual consultations. They lend support to the cautious position recommended for Doctors and patients when considering the appropriateness and safety of video consultations, which should involve willingness to move to in-person consultations if the need arises [43].

As in other pandemic caregiver studies, inability to accompany patients to appointments and be included in important medical consultations negatively impacted caregiver perceived efficacy [23, 24, 26], and caused feelings of sadness, guilt and regret. These emotions related not only to their inability to offer emotional support, but also practical support in terms of keeping check on the care and treatment provided, with caregivers left feeling helpless and experiencing loss of control, particularly when mistakes or poor treatment occurred. This points to the important advocacy role fulfilled by informal caregivers in healthcare settings during ‘normal’ times [38, 46], whilst also suggesting the greater need for caregiver involvement during times of crisis and strain within the healthcare system, when mistakes are more likely to be made.

### Differences across care-settings

There were clear patterns in the quality of care and communication received across settings. Many people opted for home-deaths over institutional admission to remain together. Corroborating our quantitative results [14], participants were more satisfied with community palliative care, with the most positive experiences involving cancer and palliative care charities providing crucial care and support face-to-face. Restrictions on visiting homes likely amplified the appreciation for these services, which participants felt went ‘the extra mile’. Community care providers were more likely than their hospital counterparts to keep participants well informed and were more easily contactable, which was especially important for relatives acting as carers. They were also perceived as the most compassionate listeners, perhaps as these interactions were most likely to be face-to-face. Few people described negative community care experiences; those that did typically involved inaccessibility of GP appointments, insufficient hospice places or lack of district nurses. This highlights the value of person-centred, community-based care models during pandemics.

Hospitals were most frequently associated with negative experiences. Although there were valued examples of compassionate care from hospital staff, participants commonly described sub-standard care and communication, reflecting the overwhelming impact of the pandemic on NHS inpatient settings [47, 48]. In cases of care transfer (e.g. hospital to hospice), satisfaction generally increased following hospital discharge. This demonstrates the benefits of specialist, hospice based EoL care, which has been consistently linked with higher satisfaction and reduced distress among those bereaved [19, 32, 49].

### Strengths, limitations and implications for further research

This study benefits from a relatively large sample size for a qualitative dataset and the combination of free text survey responses and interview transcripts which allows both a broad and in-depth exploration of experiences. Themes were largely derived from survey analysis, with the more detailed accounts given in interviews confirming and expanding on these themes. Although the survey responses were in themselves rich, focused and insightful [32], the interview data allowed the idiosyncrasy and nuances of individual journeys to be better appreciated, demonstrating that alongside commonalities, individual experiences were unique and significantly influenced by social, familial, and professional situations.

Limitations of the study include selection bias due to convenience sampling. Of those bereaved, those with stronger opinions may have been more inclined to respond to the survey than those whose felt their EoL experiences were unaffected by COVID-19. The experiences described may also be more negative than studies involving caregivers of cancer survivors (e.g. 39). The voices of men and people from ethnic minority backgrounds are underrepresented in the survey data in particular, which may result in gender and cultural bias. Further research should focus on exploring the perspectives and caregiving experiences of men and people from ethnic minority backgrounds.

### Implications for policy and practice

This study has important service provision and policy implications. In our emerging post-pandemic world, it is vital that health and social care institutions, and the professionals working in them, learn from the experiences of people bereaved during the pandemic to rebuild integrated, well-resourced, compassionate cancer services, whilst also considering preparedness for future similar events.

At a policy level, clearer guidance for exceptional circumstances such as EoL and bereavement in future pandemic-like events would reduce the confusion and guilt experienced by many people who found the inconsistent, conflicting messages highly distressing. Hospitals and other care settings should consider the emotional needs of patients and their loved ones at EoL when determining hospital policy, e.g. of general visiting hours in hospitals for patients receiving EoL care both in future pandemic and current non-pandemic times. This should include better planning on how to maintain access to essential services during times of crisis, and debate over whether termination of treatment and exclusion of caregivers from in-person appointments is necessary or justified during such times.

Greater investment into palliative care within the NHS is also clearly needed to increase the capacity of specialist teams in acute and community settings. This would help reduce disparities in experiences of EoL care in hospital versus in the community or a hospice, whilst also sustaining access to highly valued community nursing and hospice services which can be left vulnerable if overly reliant on charitable funding. Investment and training is also needed to improve generalist palliative care skills and competencies in all community and hospital staff involved in palliative and EoL care, thereby reducing some of the demand on specialist palliative care services [50, 51]. Specialist services could then play a major role as advisors and educators of generalist health and social care staff, providing direct care for the more acute or complex cases. Personal, familial, and cultural values impact views on medication, care setting and care priorities, all of which should be elicited and taken into account in holistic, shared decision-making [52, 53]. High quality, holistic, compassionate care should be prioritised for all cancer patients in mainstream or specialist centres, with an increased emphasis on regular, clear and compassionate communication with family, friends and chosen family as a priority in EoL settings [19, 50, 54]. Given the increased use of these methods post-pandemic, and in preparedness for future pandemics, specific consideration should be given to ways of achieving this using virtual communication methods, as well as other ways of maintaining effective and compassionate communication during pandemic or other emergency situations.

## Conclusion

The end of life and early bereavement are challenging, emotional times, which were distorted and amplified during the pandemic. With disruptions to care delivery, specialist services and availability of support, COVID-19 negatively affected care experiences of people bereaved due to cancer across the UK. Social restrictions, particularly for those shielding, significantly hindered people’s ability to provide and receive usual support, with many regretting the loss of time spent together. COVID-19 was perceived to dominate the NHS, with infection control prioritised over caring for those with chronic, terminal conditions, often leading to poor quality care which did not meet family expectations. The disruption to cancer services added to people’s doubts that their relative would have died under non-COVID circumstances, with many reporting the death as ‘unexpected’ – a strong predictor of symptoms of prolonged grief disorder [15]. As services re-build post-pandemic, improvements in palliative care in hospitals, investment into community care, and ensuring compassionate communication with patients and families must be prioritised, alongside developing preparedness in our healthcare systems for future pandemics or large-scale outbreaks of infectious disease.

## Electronic Supplementary Material

Below is the link to the electronic supplementary material.

Supplementary Material 1

Supplementary Material 2

## Data Availability

The datasets presented in this study can be found in online repositories. Harrop E, Selman LE. Bereavement During COVID-19 in the UK: A Mixed-methods Study of the Experiences of Bereaved People and Bereavement Services, 2020-2022. UK Data Service via https://reshare.ukdataservice.ac.uk/855751.
