# Supplementary material for: “I have never felt so alone and vulnerable” – A qualitative study of bereaved people’s experiences of end-of-life cancer care during the Covid-19 pandemic
Source: BMC Palliat Care. 2024 Dec 26;23:300. doi: 10.1186/s12904-024-01619-9 (PMC11670489; doi:10.1186/s12904-024-01619-9)
Supplement: Supplementary file 2 — Supplementary Material 2 [file 12904_2024_1619_MOESM2_ESM.docx]

**Interview topic guide; Interview One**

**Study title:** The grief experiences and support needs of people bereaved during the COVID 19 pandemic: semi-structured interviews

N.B This guide will be adapted through piloting and as data collection progresses, in discussion with the research team and advisors, to explore perspectives and patterns in the dataset.

**Administration:** Researcher to thank the participant for taking the time to talk to us, refer to the information previously provided to them, outline what the interview will cover, how long is likely to last, that will be picking up on some of answers given in the questionnaire to explore in more depth, that will record with their permission, anonymisation, that they don’t need to answer any questions they don’t feel comfortable with, can stop the interview at any point etc. If they have a companion with them who has not completed a consent form, seek verbal consent after the recording has started agreeing to the recording and that we use their data as outlined in the information sheet. Also make note of who they are in relation to participant.

In general, do not suggest responses. If a respondent has expressed difficult understanding a question or prompt it may be necessary to offer a brief example to clarify and illustrate. However, this should be done after the respondent has been given ample opportunity to answer spontaneously.

Please ensure you are familiar with the person’s survey responses before you begin. If there are specific areas mentioned e.g. end of life care experiences, unmet support needs, barriers to accessing support, please note these down for exploration/reference in the interview.

**Example Introduction:** Thank you for agreeing to speak to me today. *I am going to ask you about the questionnaires you filled out. First I’ll ask you tell me a little bit more about the your grief experience. Then I’ll ask how that event may have affected you and your family during the pandemic and what (if any) support you access or were provided. In general, I don’t need a lot of information just enough so I can understand your experiences and any problems you may have had. The interview will be between 30-45 minutes. Please let me know if you find yourself becoming upset as we go through the questions so we can slow down and talk about it. You don’t have to answer any questions you are not comfortable with you can take a break at any time. Also, let me know if you have any questions or don’t understand something.*

*Do you have any questions before we start?*

**Interview guide**

**Context**

- In your questionnaire you told us *(brief reference to EoL experience if relevant)*

Would you like to tell us a little more about what happened and the care your ….. and you and your family received? *(Prompts: Do you know whether or not specialist palliative care professionals were involved? What care or support did they give your …… and you/your family? How well supported did you feel by the health care team around the time that they died?)*

- What (if anything) helped you around this time? *(prompt: Any elements of good practice from care home, hospice or hospital).*

**More detailed exploration of impact of this loss**

- How did this effect you and your family? *(prompts: at the time, more recently, how family as a whole are coping).*
- In your Q you *agreed/disagreed* etc with the statement *'I am able to face the pain which comes with loss’.* Do you still feel this way?
- Can you tell us more about this feeling? (prompt: What do you see as the main reasons for feeling like this?)

**Focussing on the key areas (bereavement during a pandemic)**

- Are there any other ways that you feel the pandemic has affected your mourning or grieving experience? *(prompts: funerals, social distancing measures, media coverage, social media, ongoing threat of virus inc. others behaviours and attitudes to it)*
- How did you and others in your family cope with these difficult sets of circumstances?
- Were there any other circumstances or challenges that affected you during this time? (prompt as appropriate; economic/financial, working conditions, school closure, wider community impacts e.g places of worship, other community resources/centres)
- In your Q you *agreed/disagreed* with the statement *'It may not always feel like it, but I do believe that I will come through this experience of grief.*' Do you still feel this way and can you tell us more about this feeling?
- What has helped or would help you to cope better with your grief & bereavement at this time? (prompt; is there anything in particular that is making it difficult for you to cope?)

**Pulling out the essential points (accessing support)**

- You've mentioned the support that you've received from *(……survey & interview if mentioned already-include informal support here too).* Has any of this changed since you completed the survey? (e,g are you now accessing formal bereavement support?
- What have been the main ways in which you have been helped by this support?
- Are there any aspects of this support that have been difficult or could have been better? *(prompt with regard to quality of support, mode of delivery and access to it- reference survey answers where relevant too)*
- Is there any other form of support you would like to be getting? (prompt if relevant; What is stopping you from getting this?)

**Conclusion**

- What have been the most difficult aspects of losing someone during a pandemic? (prompt: how has it compared to previous losses of close family or friends? How have these previous bereavements affected your current experience and ability to cope?)
- What (if anything) has really helped you at this time?
- How could support for families be improved at the end of life, in early bereavement or the months that follow?
- Is there anything else you would like to mention to us?
